# Electric vehicle sound stimuli data and enhancements

**DOI:** 10.1016/j.dib.2018.10.074

**Published:** 2018-11-02

**Authors:** D.J. Swart, A. Bekker, J. Bienert

**Affiliations:** a Stellenbosch University, South Africa; b Technische Hochschule Ingolstadt, Germany

## Abstract

Data for six electric vehicle WOT interior sound measurements and eight enhanced sound signatures are presented. The measurement of electric vehicle interior sound signature data and the enhancement of these stimuli are documented in this data article. The procedures and equipment that were used to record the data, as well as the transposition, harmony and order addition, frequency filtering and modulation enhancement techniques that were applied to these stimuli are explained in detail. The transient frequency content of the 12 sound stimuli is presented in acoustic spectrograms along with the audio files in.mp3 format.

**Specifications table**

**Table:** 

Subject area	*Engineering*
More specific subject area	*Automotive Acoustics, Psychoacoustics*
Type of data	*Table, figure and sound files*
How data were acquired	*Interior sound measurements through a Head Acoustics BHS I binaural headset.*
Data format	*Raw and enhanced*
	*Example:*
	*Sound A.mp3 – Original interior Wide Open Throttle sound measurement.*
	*Channel 1 (Left) – The interior sound pressure measurement at the left ear position in the driver׳s seat.*
	*Channel 2 (Right) – The interior sound pressure measurement at the right ear position in the driver׳s seat.*
Experimental factors	*The sound in the interior of the vehicle was acquired using a Squadriga mobile front end, which passes the analog voltage signal from the microphones through a analog high-pass filter, where after it is converted to a digital signal. The sound signatures were further enhanced using the Audacity and GarageBand sound software, where different sound techniques and harmonics were added.*
Experimental features	*The interior WOT sound signatures of 5 electric vehicles and one hybrid electric vehicle was recorded using a binaural measurement system. Additional enhanced sound signatures were created to diversify the stimuli pool and evaluate the consumer satisfaction.*
Data source location	*The vehicles were tested in various secluded public asphalt roads in Bavaria, Germany.*
Data accessibility	*Data are published with this article*
Related Research Article	[4] D.J. *Swart and A. Bekker: ‘The Relationship Between Consumer Satisfaction and Psychoacoustics of Electric Vehicle Signature Sound’,* 2018, *Journal of Applied Acoustics, In Press*

**Value of the data**

• The Wide Open Throttle (WOT) interior sound signatures of several electric vehicles (EV׳s) and one hybrid electric vehicle are presented.
• The sound signatures include the binaural sound pressure levels as a function of time for the driver position, during WOT acceleration of the different vehicles.
• The data provide researchers with accurate binaural sound recordings of commercial electric vehicles, which can be used for future jury evaluations of electric vehicle psychoacoustics.
• Additional enhanced stimuli are presented that were created as potential future sound signatures.
• The enhanced stimuli provide industry with realistic variations in sound signatures that enrich different components of electric vehicle sound character.

## 1 Data

Six vehicle sound signatures were recorded in the interior of standard production electric/hybrid electric vehicles on public secluded asphalt roads. The measurements were opportunistic and vehicles were assessed during test drives from vehicle dealerships. The sound signatures were acquired during WOT acceleration, which involves accelerating the vehicle from rest to a maximum speed of 120 km/h in the shortest time possible (Avg. 18.7 s). WOT acceleration provokes the maximum response of the electric motor and vehicle drive-train, which augments different aspects of the vehicle sound character during the run-up.

## 2 Experimental design, materials and methods

### 2.1 Standard production EV stimuli

**Fig. 1:**
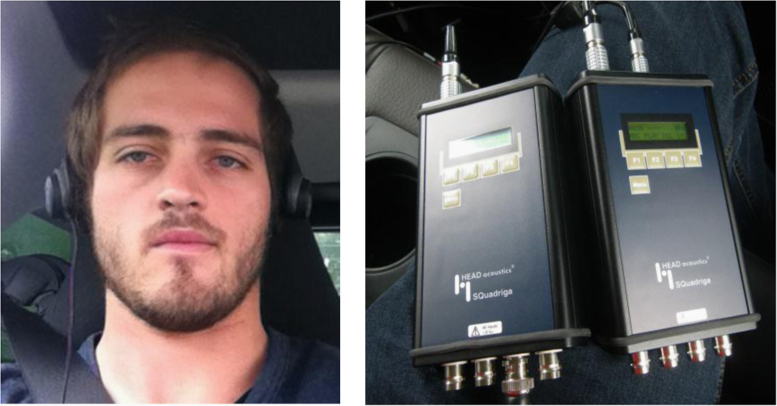
Experimental setup including the binaural BHS I headset (left) and the Squadriga I measurement system (right).

Six standard production EV/HEV׳s were evaluated with details as shown in Table 1. The vehicle specifications, driving conditions and locations are, respectively, indicated. The stimuli were recorded under WOT driving conditions (maximum acceleration) from a position of rest to a maximum speed of 120 km/h. The measurements were repeated a minimum of four times in both driving directions depending on the available time and test conditions (traffic, weather, etc). The interior sound stimuli were recorded in the driver seat of the vehicle using a Squadriga I data acquisition system from Head Acoustics [5] and a BHS I binaural headset as shown in Fig. 1, using a sample rate of 44.1 kHz. The recorded runs were evaluated in the Head Acoustics Artemis Suite 5 using the FFT versus time and Level versus time analyses to assess the quality of the measurement based on external noise influences such as pass-byes and environmental noise. The best measurement was selected for each vehicle and exported to a sound file (.mp3) for jury evaluations [4].

**Table 1:** Vehicle specifications and testing locations.

**Manufacturer**	BMW	Citroën	Renault	Porsche	Smart	Volkswagen
**Model**	i3 (BEV)	C-Zero	ZOE	Panamera Hybrid	Electric (ED3)	e-Up!
**Year**	2014	2010	2010	2013	2013	2013
**Size class**	4 seater	2 seater	4 seater	4 seater	2 seater	4 seater
**Drive system**	Direct drive	Direct drive	Direct drive	Multi-stage gearbox	Direct drive	Direct drive
**Propulsion**	Electric vehicle	Electric vehicle	Electric vehicle	Hybrid electric vehicle	Electric vehicle	Electric vehicle
**Tyre make**	Bridgestone ecopia EP500	Dunlop ENSAVE 2030	Michelin green X	Unknown	Kumho ECSTA KH11	Vredestein SNOWTRAC 3
**Tyre model**	175/60R19 86Q	175/55R15 77V	195/55R16 91Q	Unknown	175/55R15 77T	165/70R14 81T
**Full run-up time**	12.5 s	19.5 s	18.7 s	24.0 s	16.0 s	21.7 s
**Temperature**	26 °C	9 °C	26 °C	20 °C	15 °C	15 °C
**Conditions**	Sunny, dry	Cloudy, dry	Sunny, dry	Cloudy, dry	Cloudy, dry	Cloudy, dry
**Location**	Interpark, Ingolstadt	Zuchering, Ingolstadt	Ochsenfeld Eichstätt	Bietigheim, Stuttgart	Interpark, Ingolstadt	Interpark, Ingolstadt
**Number of WOT runs**	4	11	6	5	5	11
**Sound file**	Sound A.mp3	Sound F.mp3	Sound C.mp3	Sound M.mp3	Sound G.mp3	Sound H.mp3

### 2.2 Enhanced EV stimuli

**Fig. 2:**
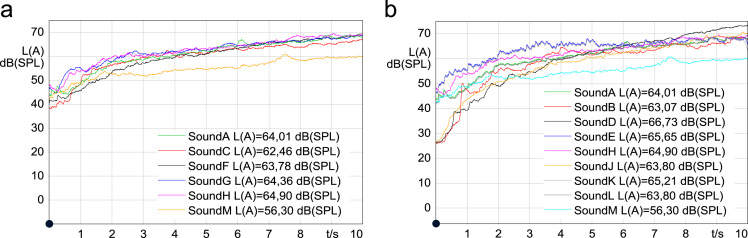
The sound pressure level versus time plots of the original stimuli (a) and the enhanced stimuli (b) with the maximum (Sound H), minimum (Sound M) and base (Sound A) stimuli included.

Several concepts were explored to add character and variation to the acoustic vehicle recordings. In the process a further six enhanced stimuli were created in order to evaluate their potential to evoke known sound quality attributes. The full description of enhanced stimuli data is provided in Table 2. Several software packages were used to generate the enhanced stimuli. Matlab was used to generate new or additive stimuli, such as side bands or harmonics. GarageBand was used to enhance the quality of the stimuli by adding reverberation effects and adjusting the sound equalization. Audacity was used to add pink noise, trim the stimuli to the desired length and amplify the stimuli to a suitable dB level. All the enhanced stimuli were amplified to fall within a 3 dB(A) absolute range (RMS) of the original vehicle stimuli in order to simulate real EV sound signatures that are level accurate. The comparison of the original and correctly amplified enhanced stimuli can be seen in Fig. 2.

**Table 2:** Details of the generated enhanced stimuli.

**Sound stimulus**	**Sound B.mp3**	**Sound D.mp3**	**Sound E.mp3**	**Sound J.mp3**	**Sound K.mp3**	**Sound L.mp3**
**Description**	1st concept sound signature	2nd concept sound signature	Computer generated stimulus from EV motor orders	Shepard-risset glissando with 110 Hz fundamental frequency	Sound E stimulus with additional pink noise	Sound A stimulus with additional modulated pink noise
**Base Stimulus**	BWM i3 interior WOT	BMW i3 interior WOT	Motor orders	Shephard׳s tone	Sound E	BWM i3 interior WOT
**Transposed**	Downwards −2400 cents	–	–	–	–	–
						
**Frequency filtering**	30 Hz	30 Hz	–	–	–	–
	(−24 dB)	(−24 dB)				
	205 Hz	205 Hz				
	(−2 dB)	(−2 dB)				
	250 Hz	250 Hz				
	(1.5 dB)	(1.5 dB)				
	1440 Hz	1440 Hz				
	(−24 dB)	(−24 dB)				
Harmony addition*	G^#^ major (f_1_ = 830.6 Hz, f_2_ = 987.8 Hz, f_3_ = 1046.5 Hz)	E major 7th (f_1_ = 659.3 Hz, f_2_ = 830.6 Hz, f_3_ = 987.8 Hz)	–	–	–	–
Order addition*	–	Lower orders (f_1_ = 150 Hz, f_2_ = 200 Hz, f_3_ = 500 Hz)	–	–	–	–
Side bands**	–	Added (f_L_ = 1100 Hz, f_U_ = 1400 Hz)	–	–	–	–
						
**Reverberation**	Added	Added	Added (44%)	–	–	–
	(44%)	(44%)				
						
**Pink noise**	–	–	–	Max level: −40.6 dB FS	Max level: −41.5 dB FS	Frequency modulated: Fm = 2 Hz, dF = 5,
						Max level: −10.1 dB FS
**Software**	Matlab, GarageBand, Audacity	Matlab, GarageBand, Audacity	Matlab, GarageBand, Audacity	Matlab, Audacity	Audacity	Matlab, Audacity

* The frequencies of the lower orders and harmonies f_1_, f_2_ and f_3_ are defined at the end of the stimulus (10 s).

** The defined frequencies are f_L_ and f_U_ are the lower and upper side band frequency values at the end of the stimuli (10 s).

Sound stimuli B and D were enhanced from the original BWM i3 stimulus (Sound A). The harmony and order additions of these two stimuli were generated in Matlab using the chirp function, sweeping from 20 Hz to the respective frequencies within the 10 s stimulus period. The addition of these enhancements can be seen in Figs. 4 and 6 respectively.

Sound stimulus E was generated in Matlab by isolating several lower electric motor orders and enhancing the stimuli with reverberation in the GarageBand software before finalizing the length and level of the stimuli in Audacity.

Sound J was created in Matlab using a Shepard׳s Risset Glissando with a fundamental frequency of 110 Hz, sample frequency of 44.1 kHz and a cycle time of 2 s [1]. The Shepard׳s tone is a set of frequency sweeps that increase linearly with time and that are specifically spaced to create an auditory illusion of a continually increasing sound [2]. These linear relationships can clearly be seen in the FFT vs time analysis of the stimuli shown in Fig. 11.

Sound K was developed in an attempt to make the enhanced sound E more realistic by adding simulated road and tyre noise. Sound l was developed to improve the warning characteristics of the base stimulus (Sound A), through the addition of frequency modulated pink noise, which improves the localization and differentiation of similar sounding stimuli [3].

### 2.3 Original and enhanced stimuli

**Fig. 3:**
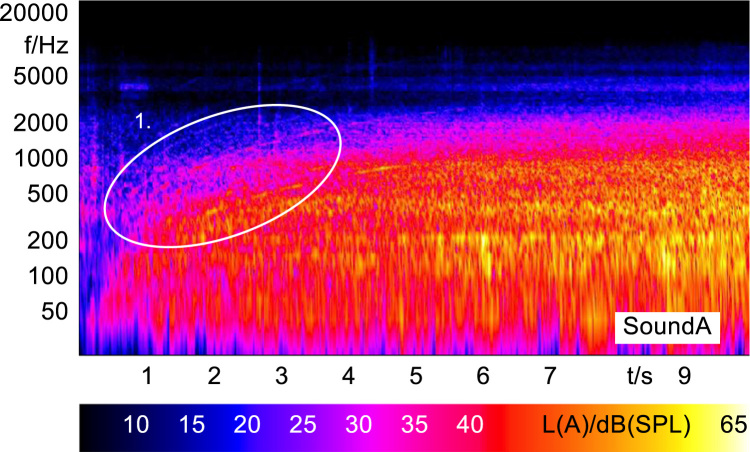
BMW i3 stimulus (original) showing the electric motor orders (1).

**Fig. 4:**
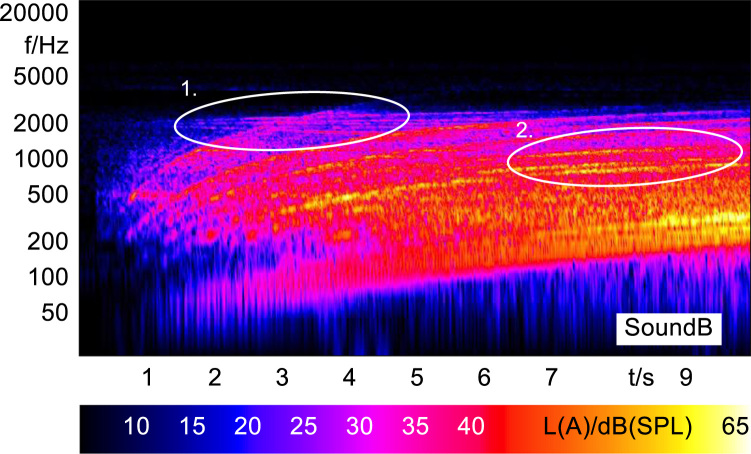
Concept 1 stimulus (enhanced) showing the transposed switching frequencies (1) and the added G^#^ harmonies (2).

**Fig. 5:**
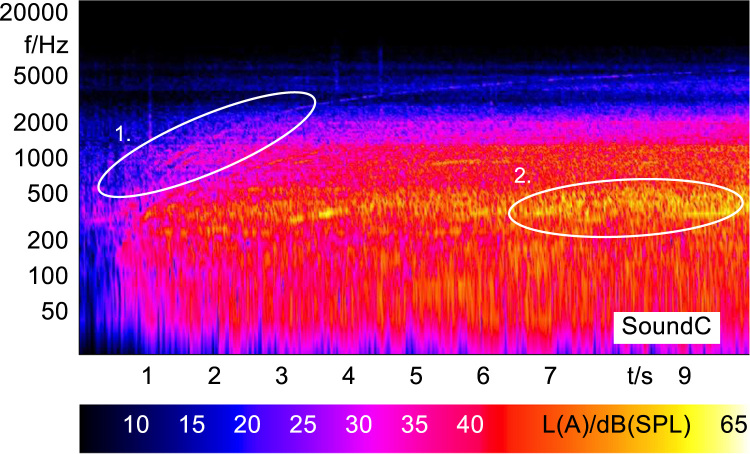
Renault ZOE stimulus (original) showing the electric motor orders (1) and the induced roughness (2).

**Fig. 6:**
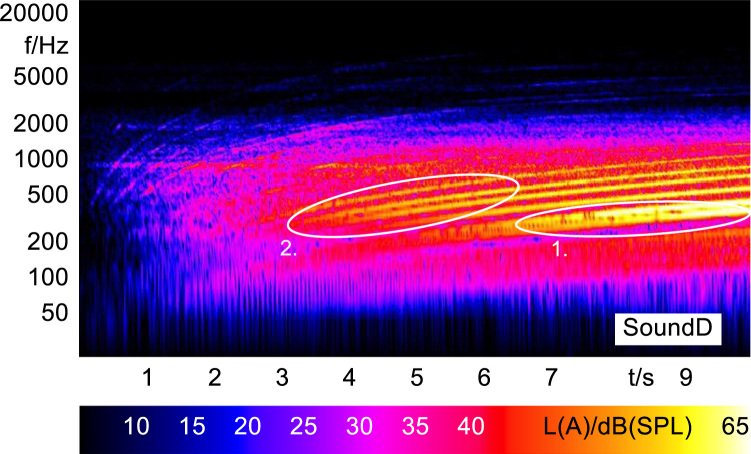
Concept 2 stimulus (enhanced) showing the addition of lower orders (1) and the E^7th^ harmonies (2).

**Fig. 7:**
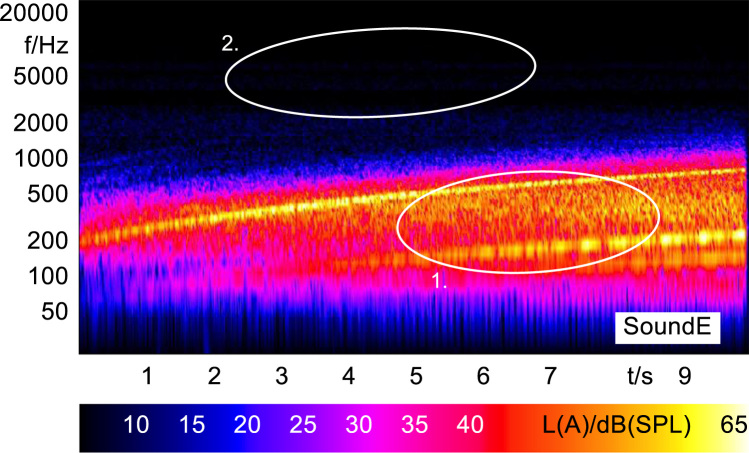
Computer stimulus (enhanced) showing the simulated motor orders (1) and the limited broadband noise in the higher frequency range (2).

**Fig. 8:**
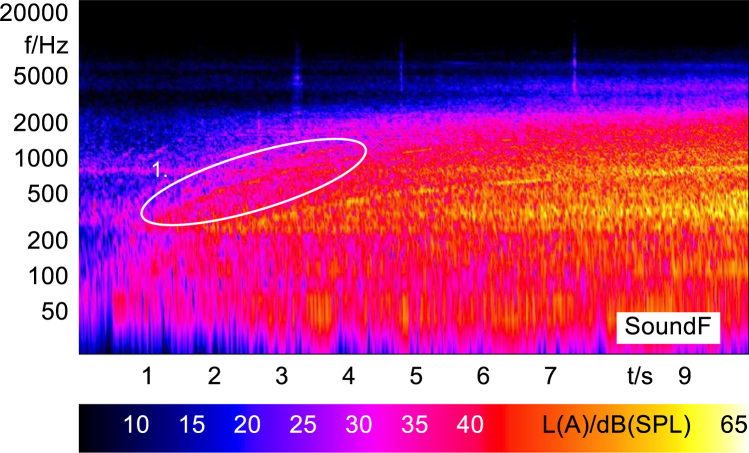
Citroën C-zero stimulus (original) showing the electric motor orders (1).

**Fig. 9:**
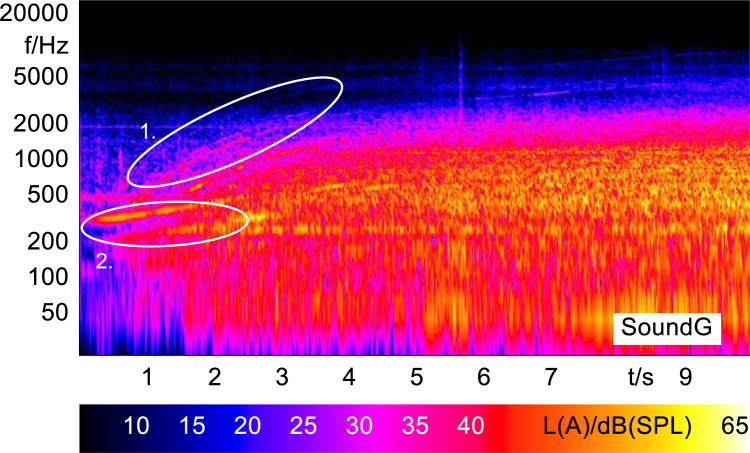
Smart electric stimulus (original) showing the electric motor orders (1) and the artificial warning sound (2).

**Fig. 10:**
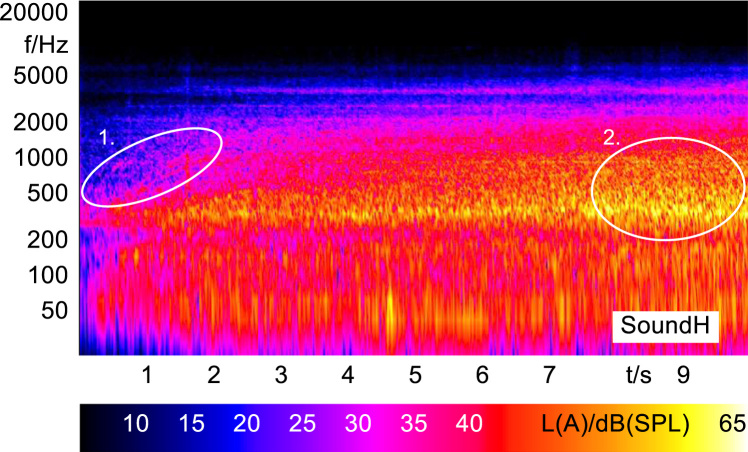
Volkswagen e-Up! stimulus (original) showing the electric motor orders (1) and the roughness induced by wind and tyre noise (2).

**Fig. 11:**
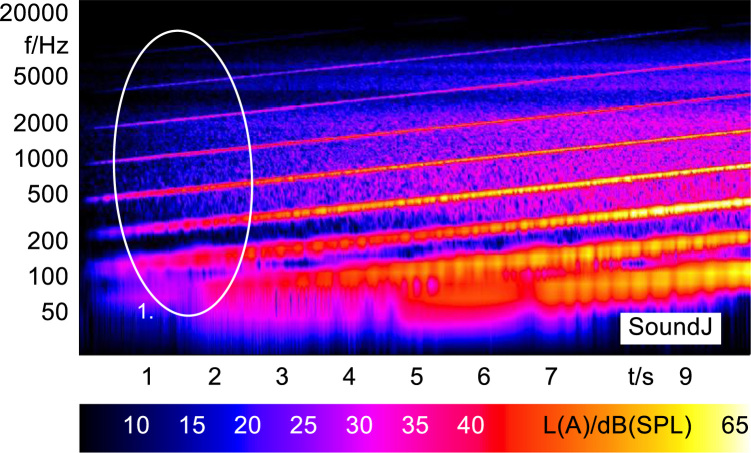
Shephard׳s tone stimulus (enhanced) showing the set of linearly increasing frequency sweeps (1).

**Fig. 12:**
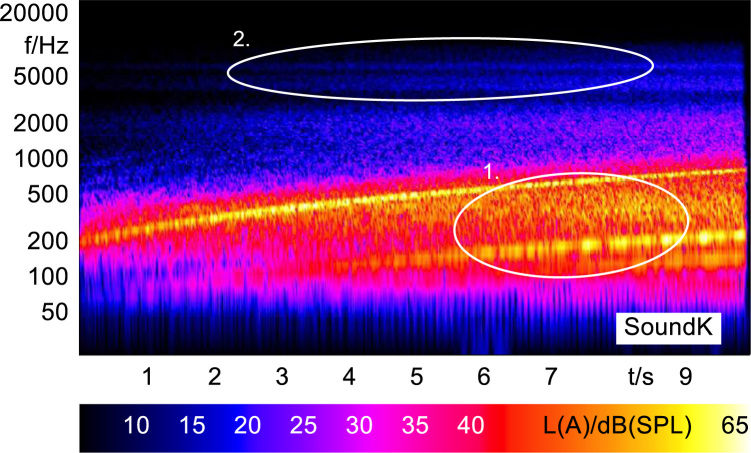
Sound E with additional pink noise (enhanced) showing the simulated motor orders (1) and the added background pink noise (2).

**Fig. 13:**
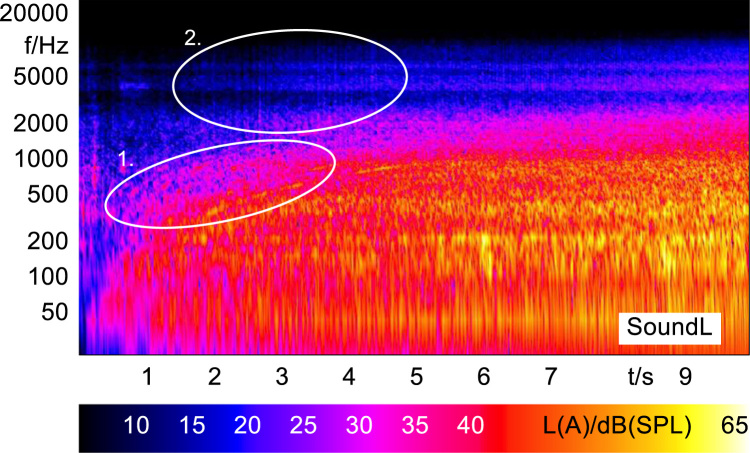
Sound a with frequency modulated pink noise (enhanced) showing the electric motor orders (1) and the raster patterns of the frequency modulated pink noise (2).

**Fig. 14:**
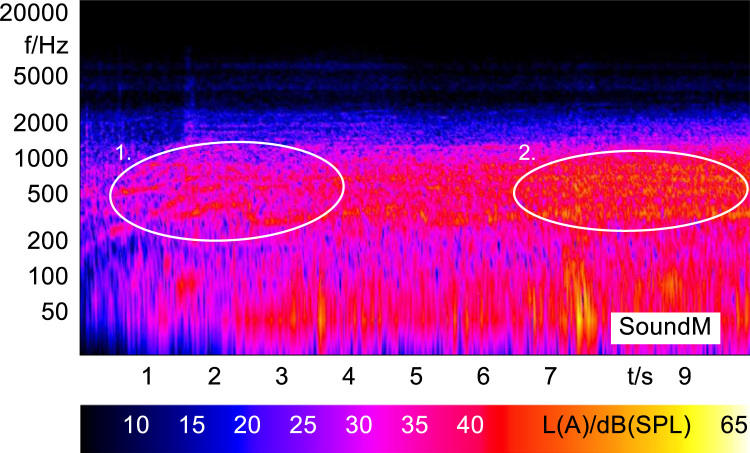
Porsche Panamera Hybrid interior WOT (original) showing the multi-stage gearbox electric motor orders (1) and the low levels of wind and tyre noise (2).

In order to analyze and visualize the differences between the sound stimuli with respect to the spectral and temporal content, spectrograms (FFT versus time) of the stimuli were created and are shown in Fig. 3, Fig. 4, Fig. 5, Fig. 6, Fig. 7, Fig. 8, Fig. 9, Fig. 10, Fig. 11, Fig. 12, Fig. 13, Fig. 14. These figures display the spectral content (ordinate) of the respective original and enhanced stimuli with respect to time (abscissa). Several sound phenomenon and enhancements are emphasized to provide an in depth explanation to the reader. The spectrograms have been widely used to illustrate sound phenomenon and attributes in vehicles sound signatures [6], [7] and [8].

## Appendix A Supplementary material

Supplementary Video 1 BMW i3 interior WOT sound signature.

.

Supplementary Video 2 1^st^ Concept sound signature.

.

Supplementary Video 3 Renault ZOE interior WOT sound signature.

.

Supplementary Video 4 2^nd^ Concept sound signature.

.

Supplementary Video 5 EV motor orders digital sound signature .

.

Supplementary Video 6 Citroën C-Zero interior WOT sound signature.

.

Supplementary Video 7 Smart Electric interior WOT sound signature.

.

Supplementary Video 8 Volkswagen e-Up! interior WOT sound signature.

.

Supplementary Video 9 Shephard’s tone sound signature.

.

Supplementary Video 10 Sound E with enhanced broadband characteristics.

.

Supplementary Video 11 Sound A with enhanced broadband characteristics.

.

Supplementary Video 12 Porsche Panamera Hybrid interior WOT sound signature.
