# Supplementary figures and images for: Electric vehicle sound stimuli data and enhancements
Source: Data Brief. 2018 Nov 2;21:1337–46. doi: 10.1016/j.dib.2018.10.074 (PMC6230979; doi:10.1016/j.dib.2018.10.074)

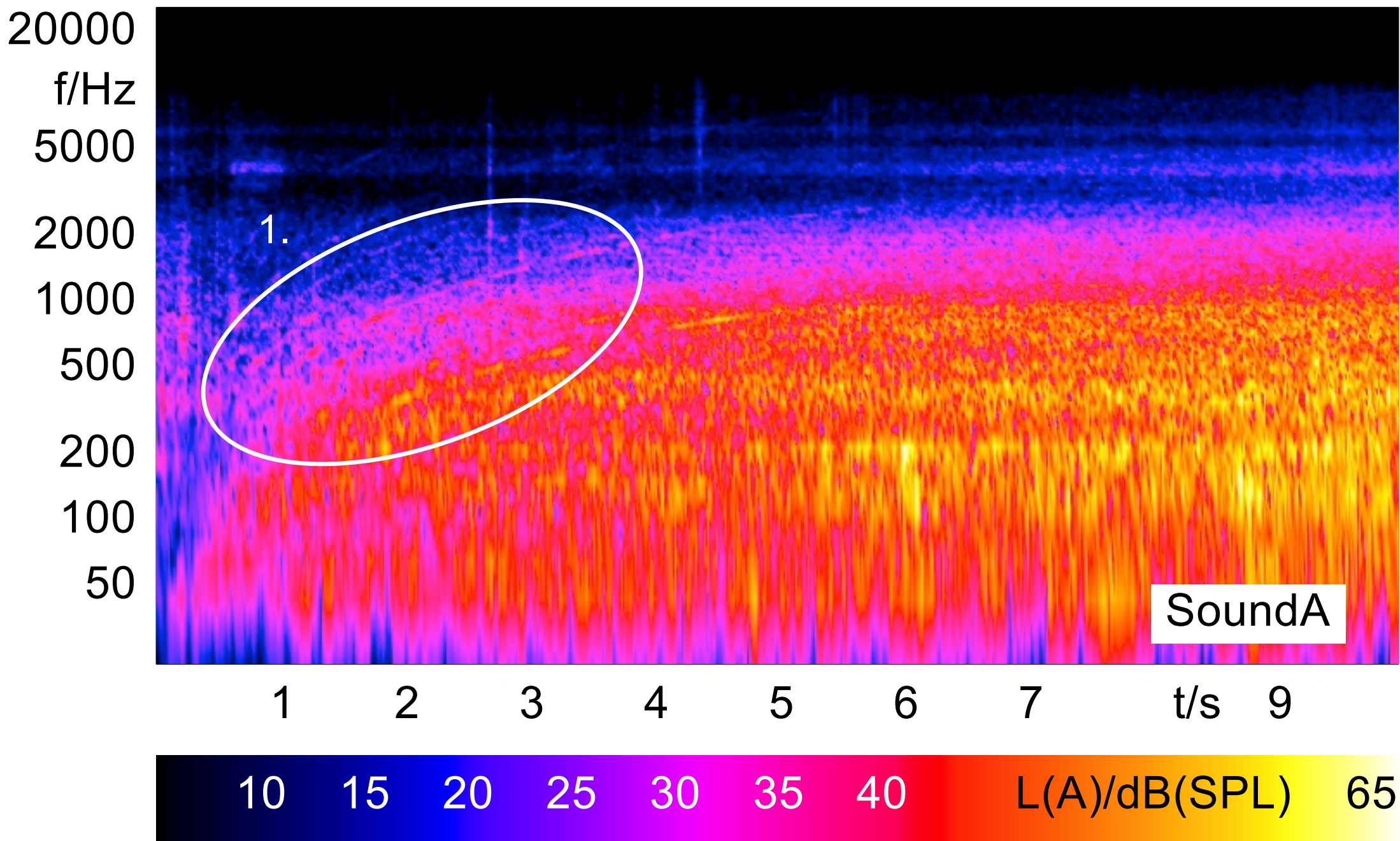

Supplement: Supplementary file 3 — Supplementary material. [file mmc3.pdf]

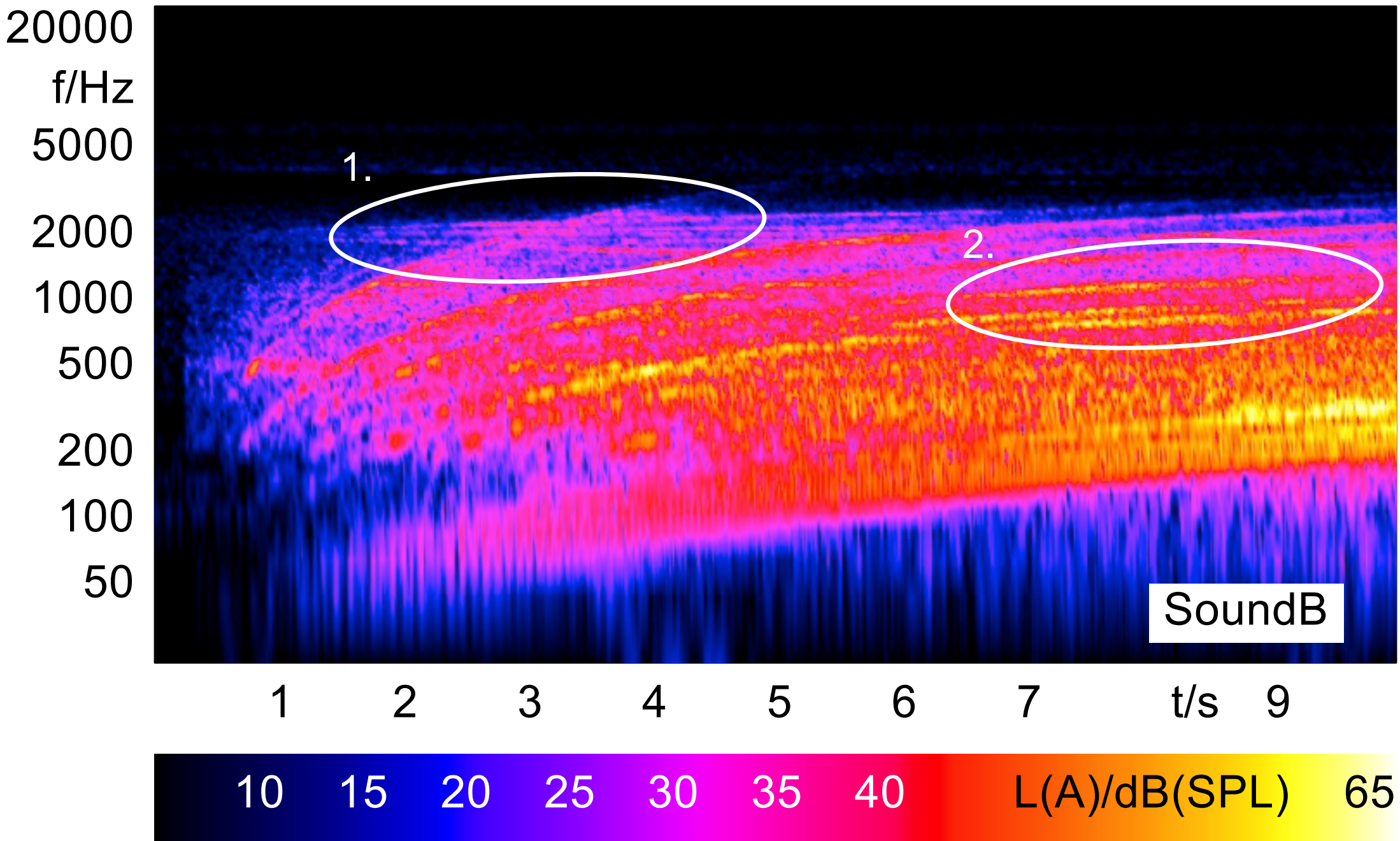

Supplement: Supplementary file 5 — Supplementary material. [file mmc5.pdf]

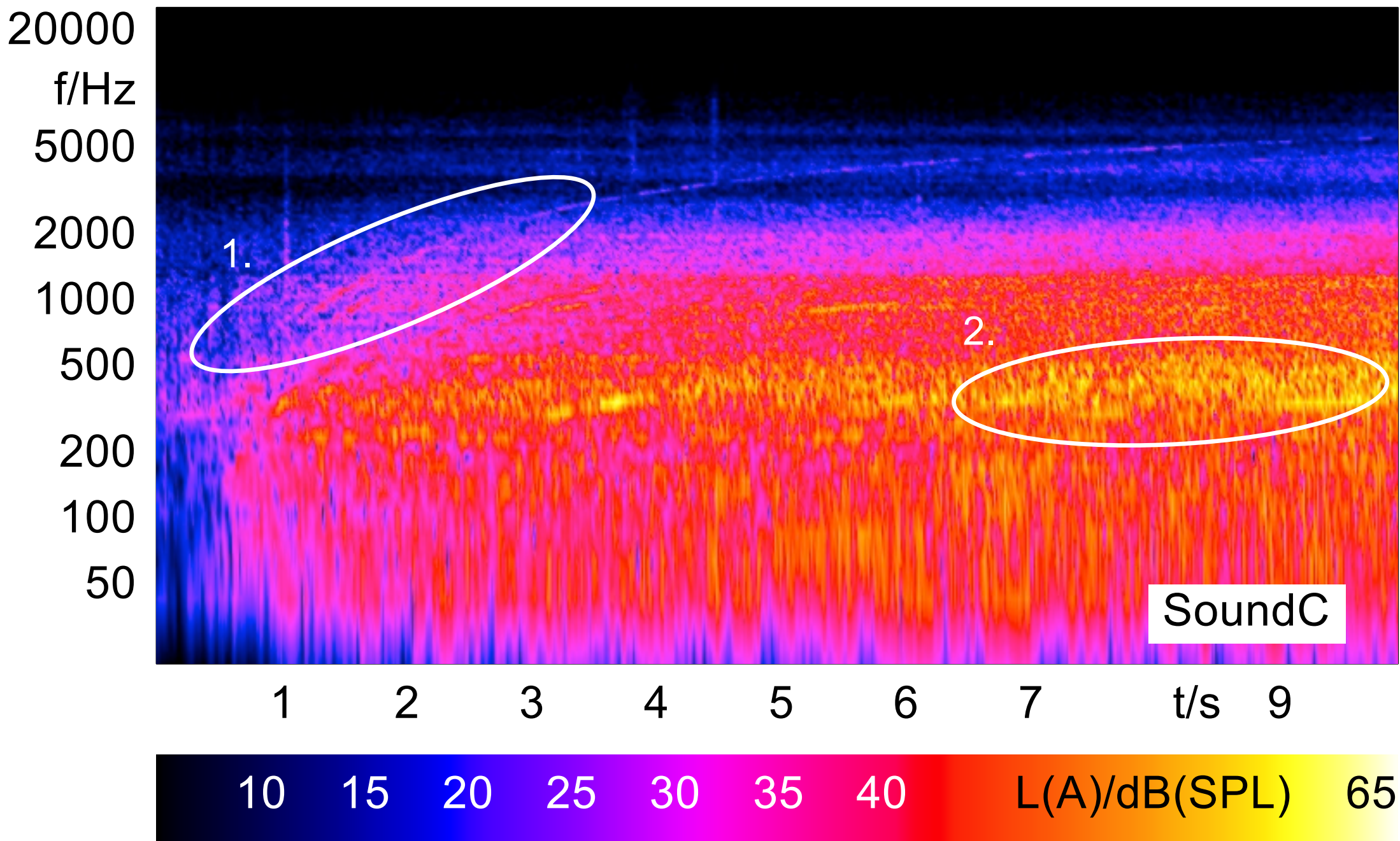

Supplement: Supplementary file 7 — Supplementary material. [file mmc7.pdf]

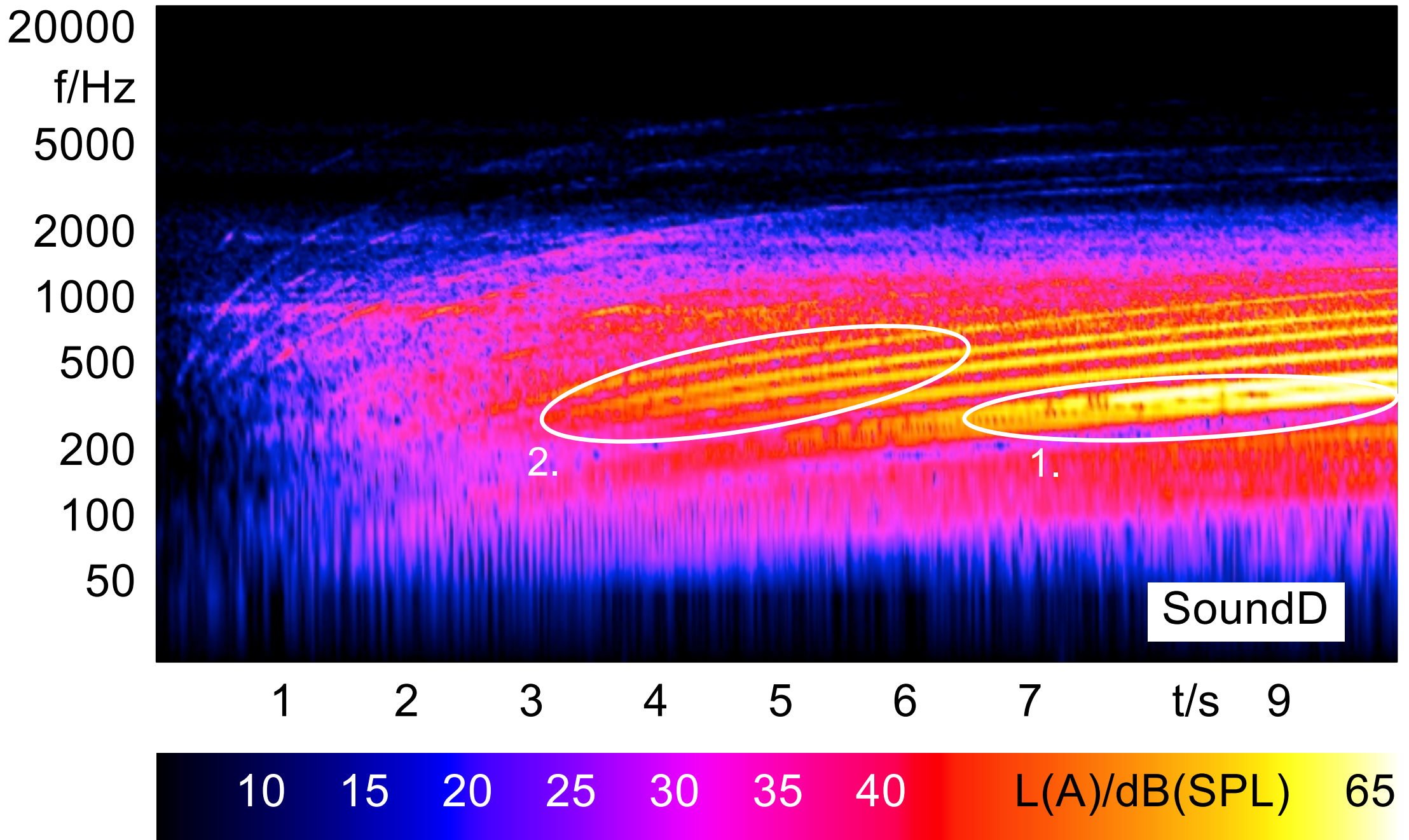

Supplement: Supplementary file 9 — Supplementary material. [file mmc9.pdf]

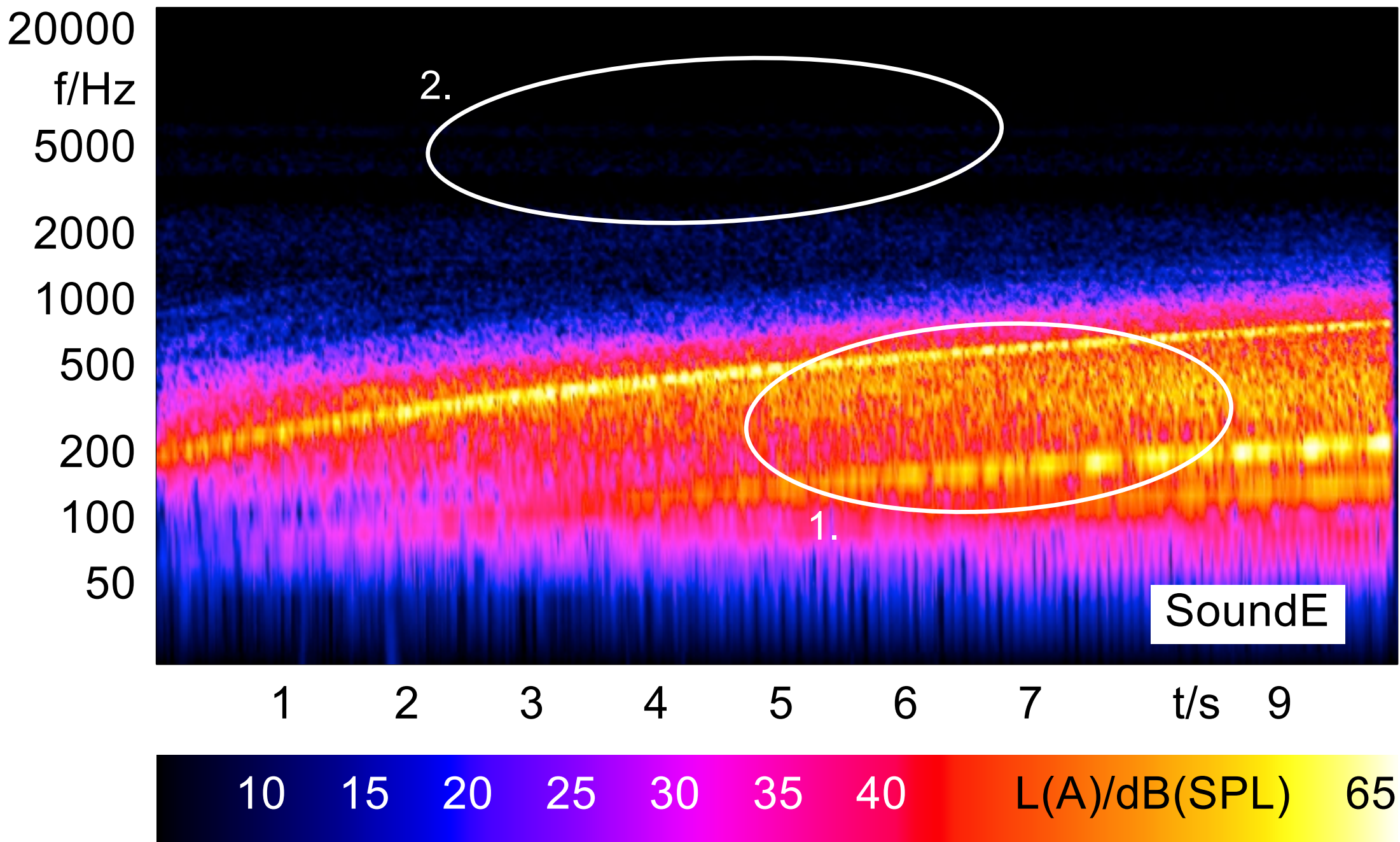

Supplement: Supplementary file 11 — Supplementary material. [file mmc11.pdf]

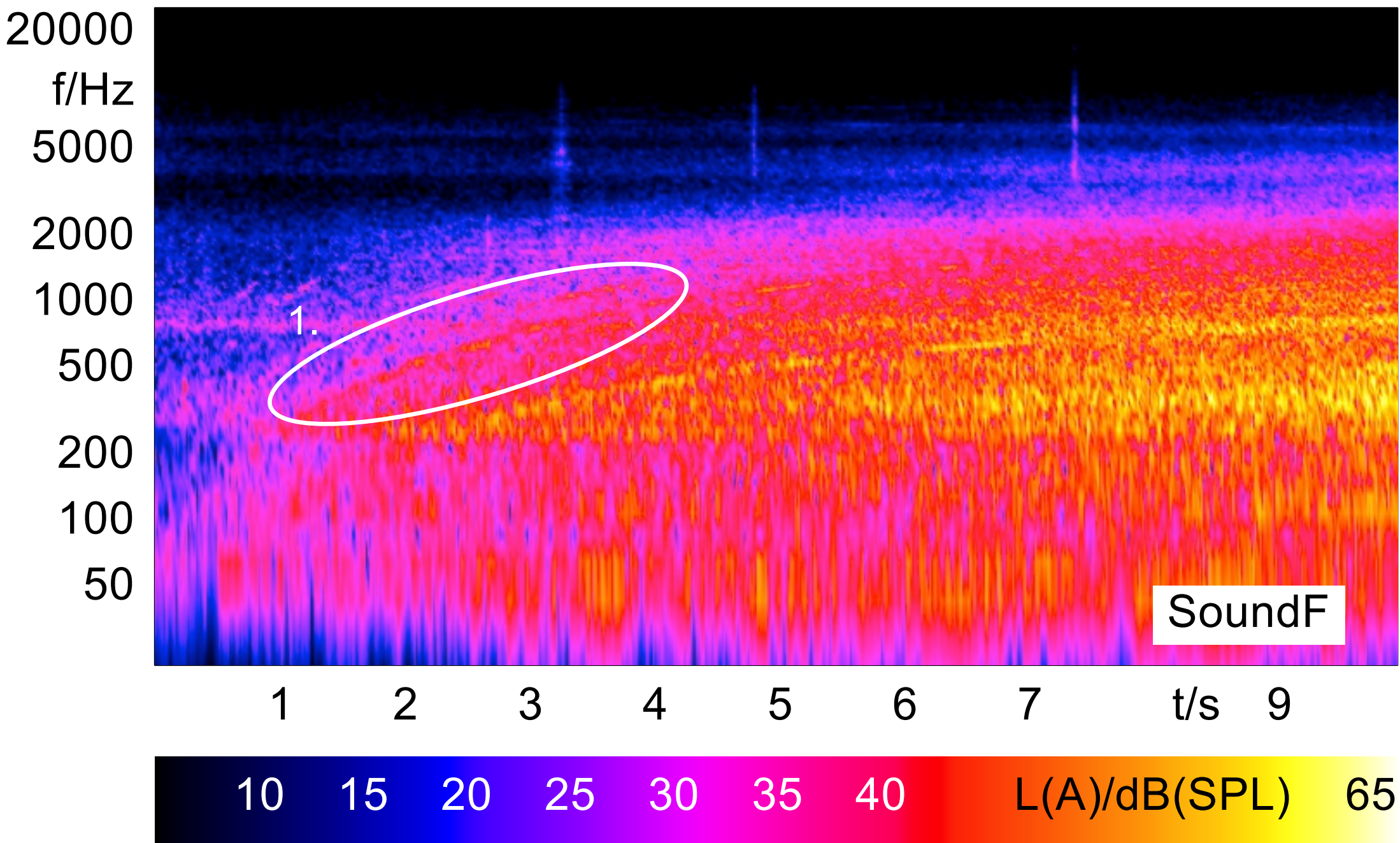

Supplement: Supplementary file 13 — Supplementary material. [file mmc13.pdf]

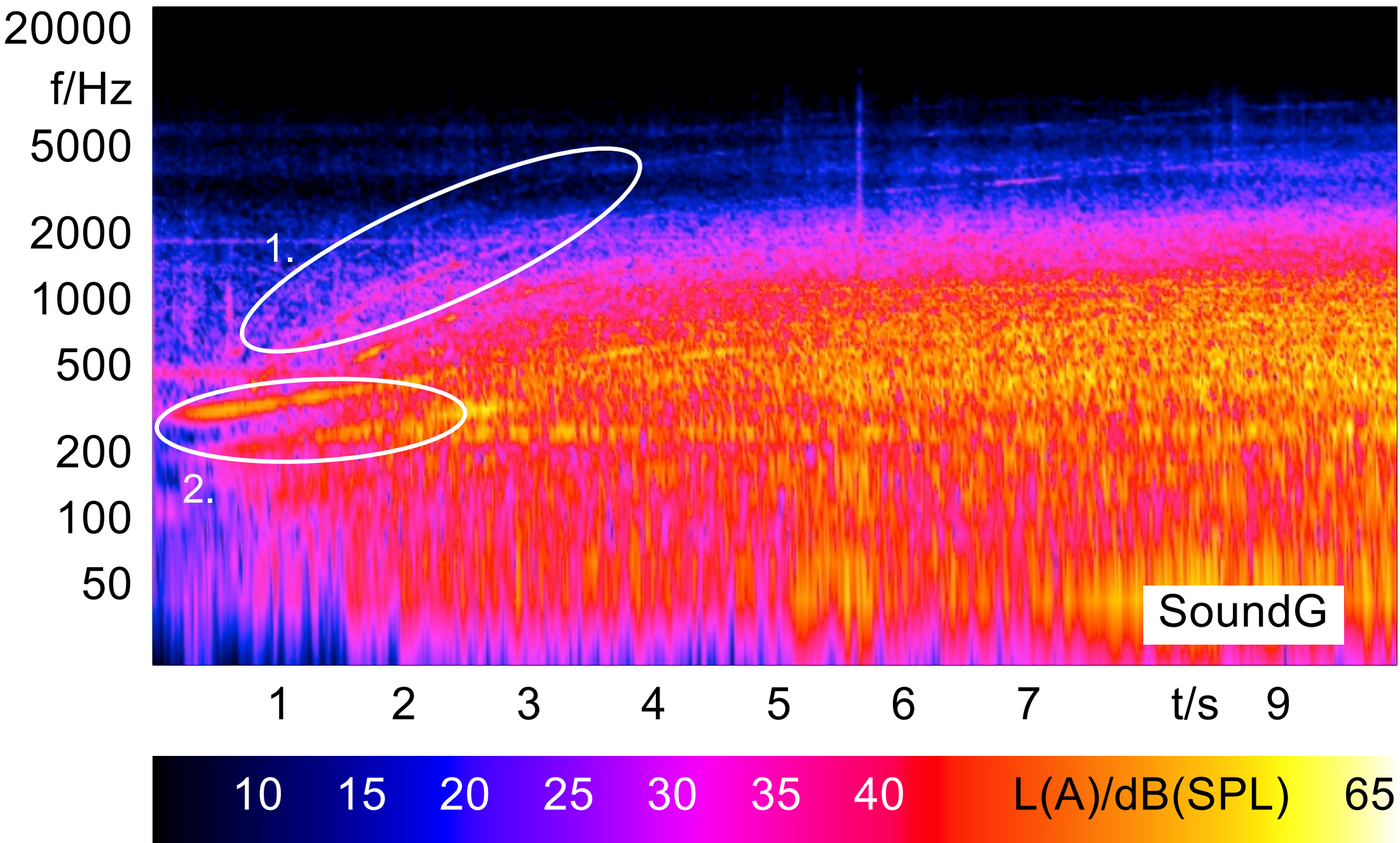

Supplement: Supplementary file 15 — Supplementary material. [file mmc15.pdf]

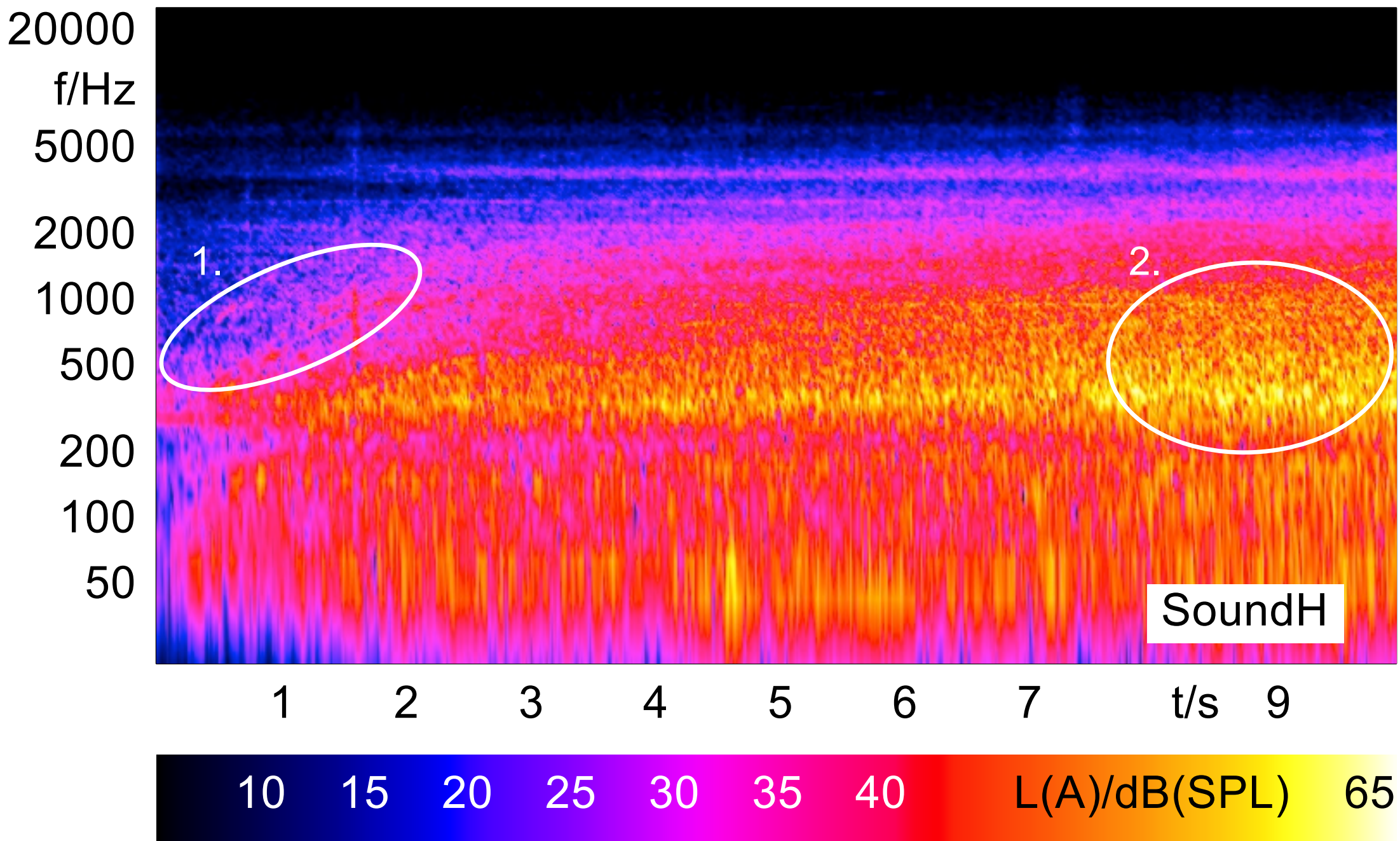

Supplement: Supplementary file 17 — Supplementary material. [file mmc17.pdf]

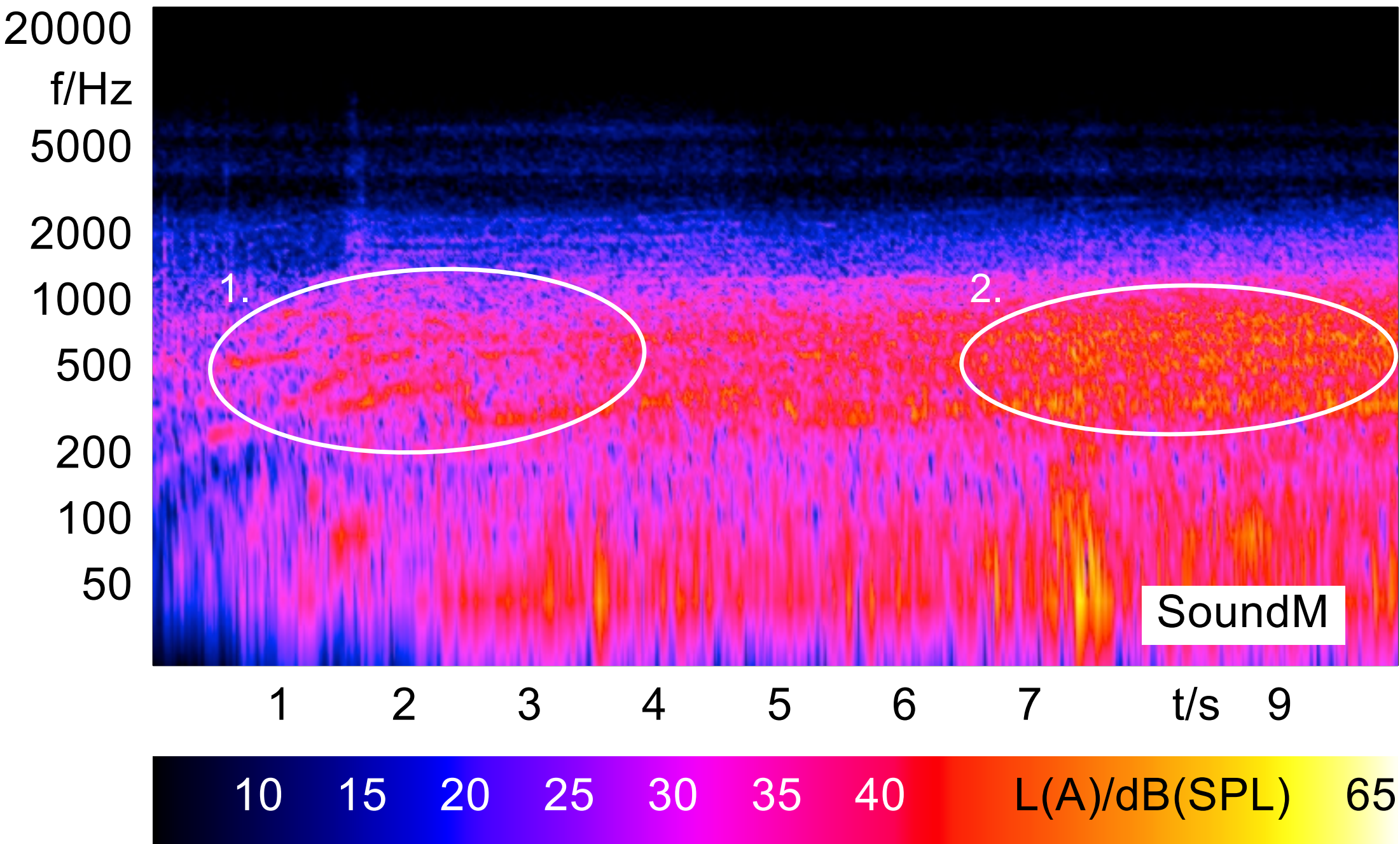

Supplement: Supplementary file 19 — Supplementary material. [file mmc19.pdf]

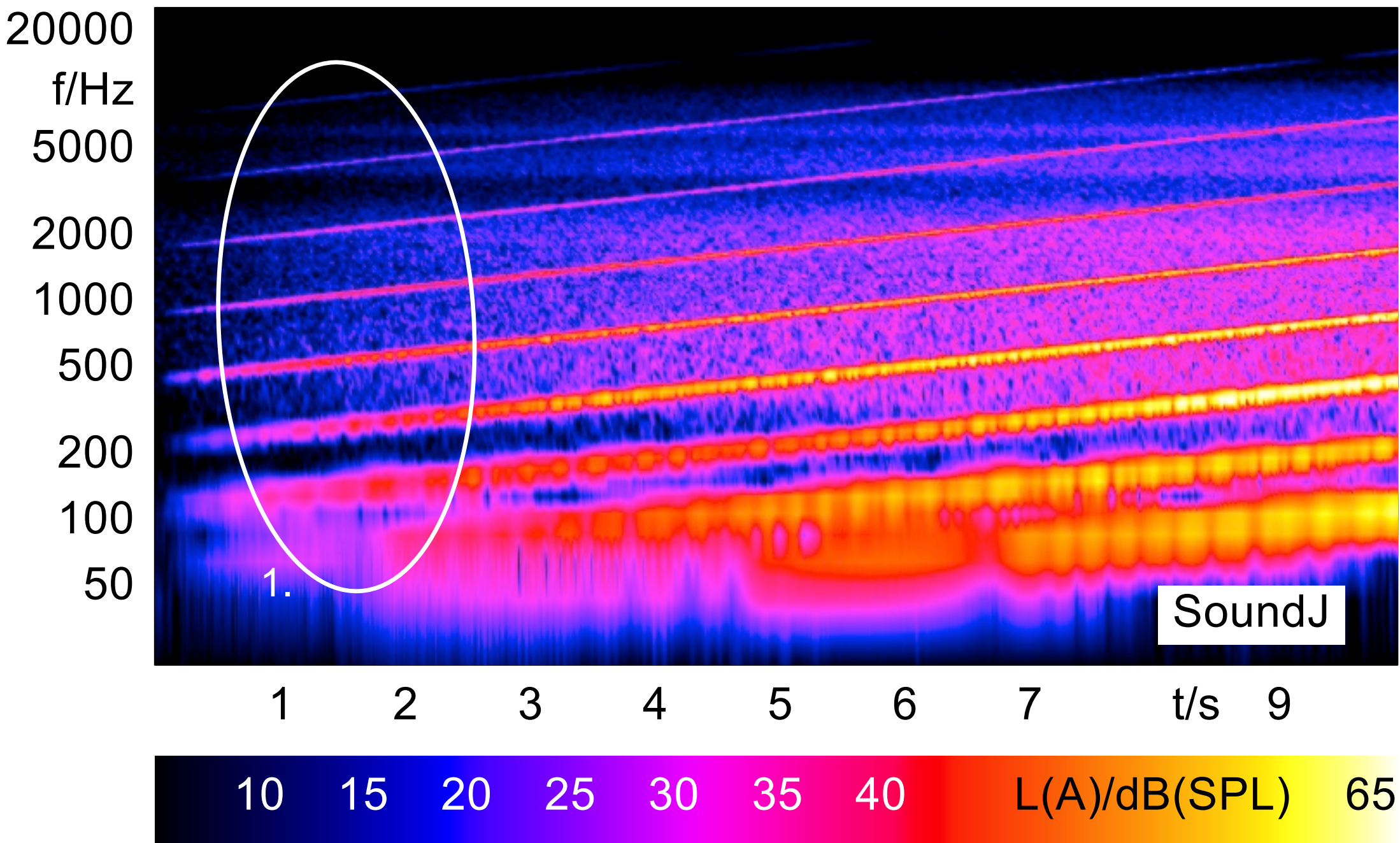

Supplement: Supplementary file 20 — Supplementary material. [file mmc20.pdf]

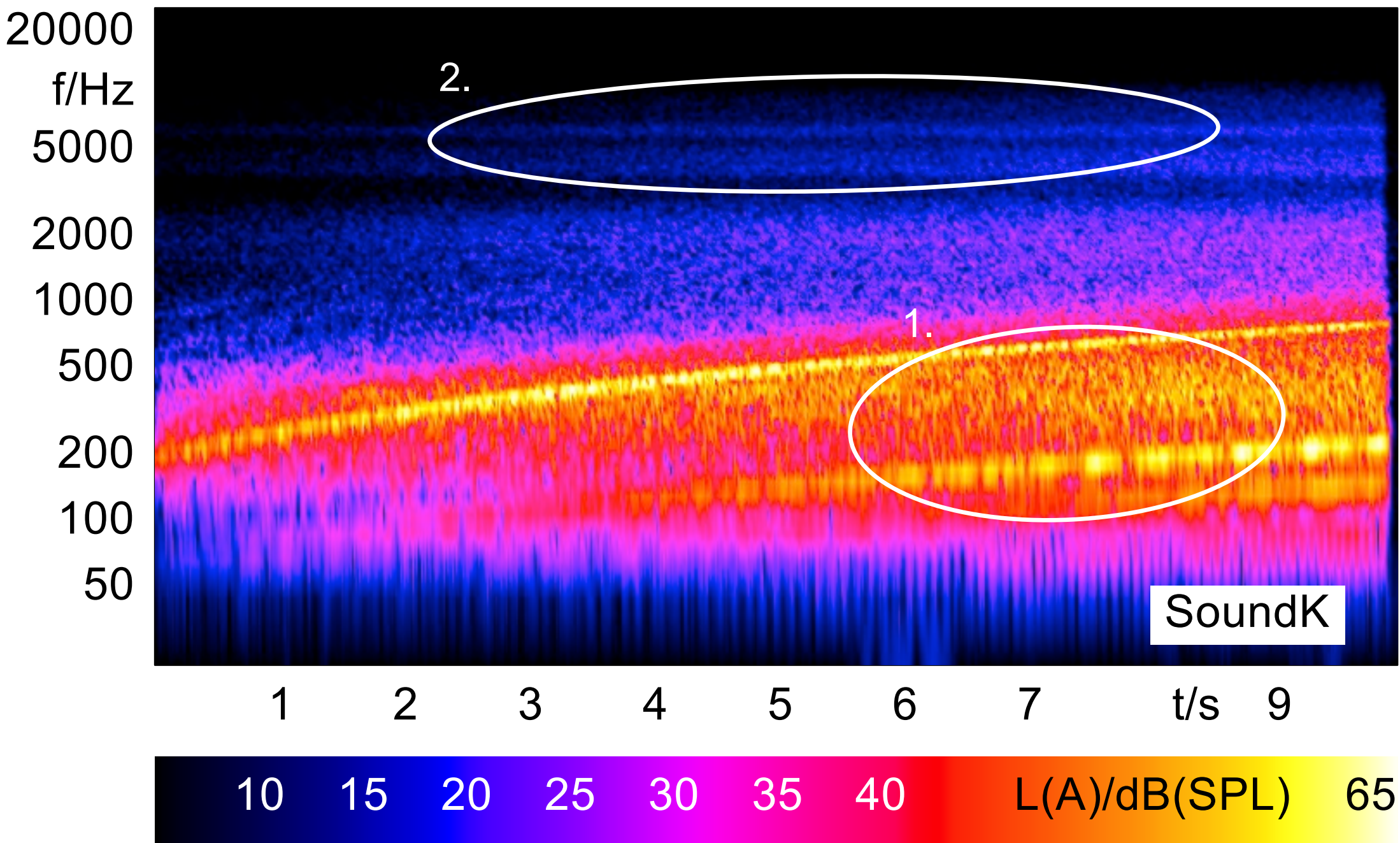

Supplement: Supplementary file 22 — Supplementary material. [file mmc22.pdf]

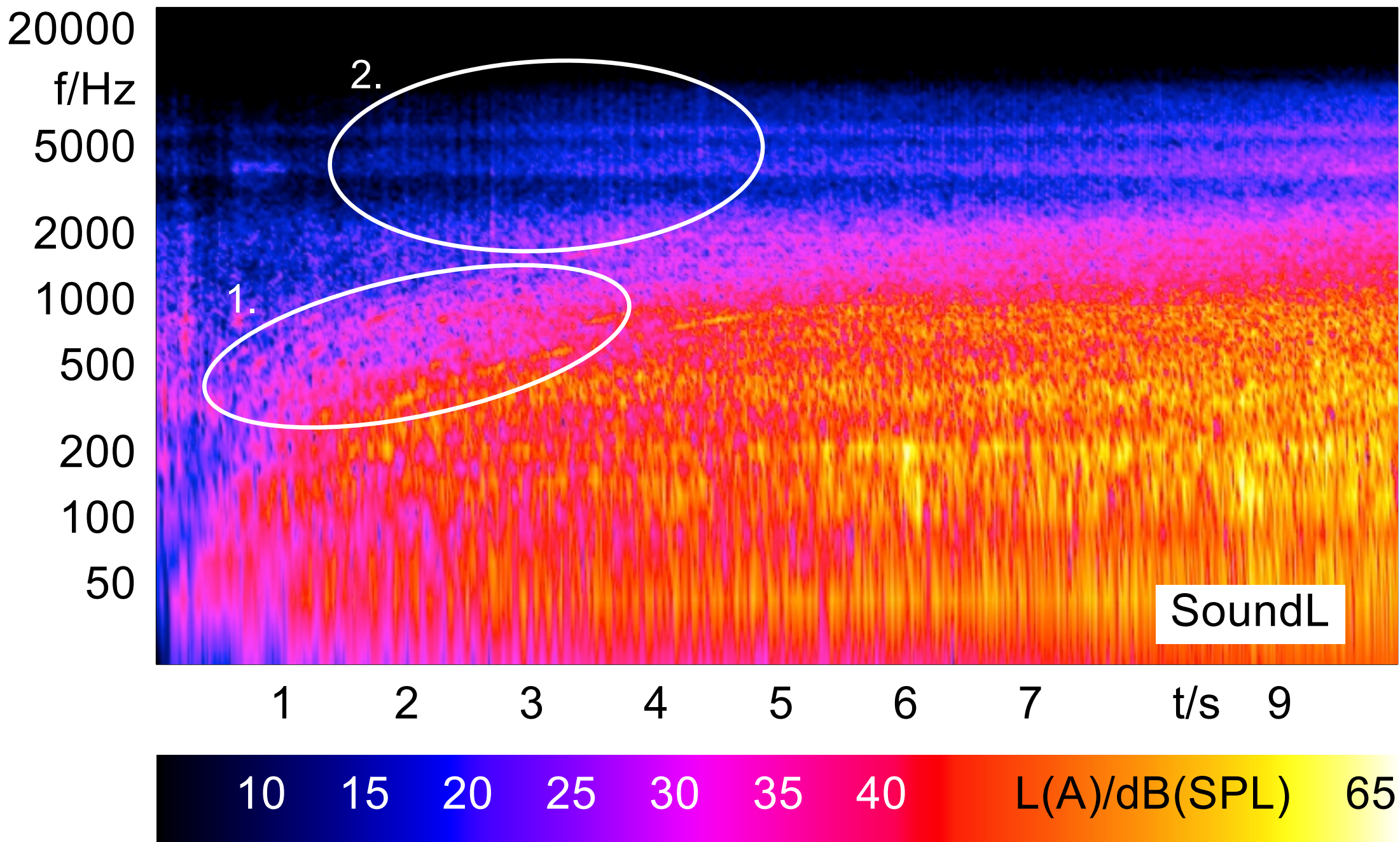

Supplement: Supplementary file 24 — Supplementary material. [file mmc24.pdf]
